# Distribution of DI*A and DI*B Allele Frequencies and Comparisons among Central Thai and Other Populations

**DOI:** 10.1371/journal.pone.0165134

**Published:** 2016-10-20

**Authors:** Oytip Nathalang, Puangpaka Panichrum, Kamphon Intharanut, Phatchira Thattanon, Siriporn Nathalang

**Affiliations:** 1 Graduate Program in Medical Technology, Faculty of Allied Health Sciences, Thammasat University, Pathumtani, Thailand; 2 Graduate Program in Biomedical Sciences, Faculty of Allied Health Sciences, Thammasat University, Pathumtani, Thailand; 3 National Blood Centre, Thai Red Cross Society, Bangkok, Thailand; Roswell Park Cancer Institute, UNITED STATES

## Abstract

Alloantibodies to the Diego (DI) blood group system, anti-Di^a^ and anti-Di^b^ are clinically significant in causing hemolytic transfusion reactions (HTRs) and hemolytic disease of the fetus and newborn (HDFN), especially in Asian populations with Mongolian ancestry. This study aimed to report the frequency of the DI*A and DI*B alleles in a Central Thai population and to compare them with those of other populations previously published. Altogether, 1,011 blood samples from unrelated healthy blood donors at the National Blood Centre, Thai Red Cross Society, Bangkok were included. Only 391 samples were tested with anti-Di^a^ by conventional tube technique. All samples were genotyped for DI*A and DI*B alleles using an in-house polymerase chain reaction with sequence-specific primer (PCR-SSP) technique. The DI phenotyping and genotyping results were in 100% concordance. The DI*A and DI*B allele frequencies among 1,011 Central Thais were 0.0183 (37/2,022) and 0.9817 (1,985/2,022), respectively. Allele frequencies were compared between Central Thai and other populations. Our data shows that DI*A and DI*B allele frequencies are similar to Southeast Asian, Brazilian, Southern Brazilian and American Native populations; whereas, these frequencies significantly differ from those reported in East Asian, Italian, Alaska Native/Aleut, Hawaiian/Pacific Islander and Filipino populations (P<0.05), corresponding to the results of a matrix of geometric genetic distances. This study confirms that the prevalence of DI*A and DI*B alleles among Central Thais is similar to Southeast Asians and different to others populations of the world. A PCR-based identification of DI genotyping should overcome some of the serological limitations in transfusion medicine and provides a complementary tool for further population-genetic studies.

## Introduction

The Diego (DI) blood group system (The International Society of Blood Transfusion, ISBT) 010 was first reported in 1955 by Layrisse et al., when anti-Di^a^ was found in the case of fatal hemolytic disease of the fetus and newborn (HDFN) [1]. Thereafter, the Diego antibodies (anti-Di^a^ and anti-Di^b^) became involved in mild and severe cases of HDFN and hemolytic transfusion reactions (HTRs) [2–9]. In general, three Diego phenotypes are found; the most common is Di(a-b+), followed by Di(a+b+) and Di(a+b-). The Di(a-b-) or Diego null phenotype is a rare phenotype found only in a child with severe hereditary spherocytosis caused by the absence of band 3 suggesting the patient represents the null phenotype [10].

Antithecal antigens (Di^a^ and Di^b^) are the products of DI*A and DI*B alleles caused by a single nucleotide polymorphism, SNP (c.2561C>T) in the human erythrocyte membrane anion-transport protein gene (*SLC4A1*). This gene encodes a substance called band 3 protein, which is expressed on the surface of red blood cells (RBCs) and plays a central role in mediating the transport of carbon dioxide in the blood. A single amino acid substitution in position 854 results in a leucine corresponding to Di^a^ antigen and proline to the Di^b^ antigen [11–13].

Because Di(b+) antigen is highly prevalent in different populations, anti-Di^a^ is more frequently found than anti-Di^b^ among pregnant women and multitransfused patients [13, 14]. Routinely, to provide compatible Di(a-) blood transfusions, both patient and donor RBCs are tested with human anti-Di^a^ using the indirect antiglobulin test (IAT). However, some limitations of serological techniques occur in patients who have previously received blood transfusions or have RBCs that give a positive direct antiglobulin test (DAT) result. Various polymerase chain reaction (PCR)-based techniques have been implemented for Diego blood group genotyping such as PCR-restriction fragment length polymorphism (PCR-RFLP), PCR with sequence-specific primer (PCR-SSP) as single or multiplex assays, real time quantitative PCR, high-resolution melting analysis and DNA microarray hybridization [15–25]. These PCR techniques used to determine DI*A and DI*B alleles are beneficial not only in transfusion medicine but also in human genetic research because the DI*A allele has been recognized as a genetic marker in biological anthropology among populations [26, 27]. The different observations of these two alleles might explain the heterogeneity and evolutionary position in populations. The purpose of this study was to report the frequency of the DI*A and DI*B alleles in the Central Thai population and to compare them with those of other populations previously published.

## Materials and Methods

### Samples

Peripheral venous blood was collected in EDTA-anticoagulated blood from 1,011 unrelated healthy Thai blood donors from the National Blood Centre, Thai Red Cross Society, Bangkok, Thailand from May to December 2015, and the study was conducted until April 2016. Written informed consent was obtained from each subject. Samples demonstrating the presence of infectious markers such as syphilis, hepatitis B and C as well as HIV were not included in this study. This study was approved by the Committee on Human Rights Related to Research Involving Human Subjects, Thammasat University, Pathumtani, Thailand. Genomic DNA was extracted from peripheral blood samples using the Genomic DNA extraction kit (REAL Genomics, RBCBioscience, Taipei, Taiwan) and then stored at -20°C until use for genotyping.

### DNA standards

Eleven known DNA samples of 1 Di(a+b-), 5 Di(a+b+) and 5 Di(a-b+) phenotypes were provided by Mr. Morakot Emthip, Histocompatibility and Immunogenetics Laboratory, National Blood Centre, Thai Red Cross Society and were used as controls. All samples were confirmed by DNA sequencing.

### Di^a^ antigen detection by IAT

Briefly, 1 drop of human anti-Di^a^ (CE-Immundiagnostika GmbH, Eschelbronn, Germany) was added to a test tube and then 1 drop of 2–3% RBC suspension in 0.9% normal saline (NSS) was added. Thereafter, one drop of 22% bovine albumin (National Blood Centre, Thai Red Cross Society, Bangkok, Thailand) was added. The test tube was mixed and incubated at 37°C for 30 min. The cells and serum mixture were washed 3 times with NSS and 2 drops of the antiglobulin serum (CE-Immundiagnostika GmbH, Eschelbronn, Germany) were added. After centrifugation, the reactions were read macroscopically and the agglutination reactions were graded as 4+, 3+, 2+, 1+ and w+. After reading the negative reaction under microscope, the IgG-coated RBCs were added to check the validity of the antiglobulin test. Negative, Di(a-b+) and positive, Di(a+b+) control cells were also tested in parallel. In addition, DAT was performed for all donor samples that gave positive results to rule out false positive results, if any. A total of 391 blood samples from Thai blood donors were tested for Di^a^ antigen phenotyping.

### Genotyping of DI*A and DI*B alleles by PCR-SSP

Genotyping of DI*A and DI*B alleles was performed by PCR-SSP technique. Primers and amplification conditions were designed. Briefly, 1 μL of genomic DNA (50 ng/μL) was amplified in a total volume of 20 μL using 5 μM of DI-AB-F forward primer 5’-GGTGTGATAGGCACTGACCC-3’, 1 μL and 5 μM DI-A-R reverse primer 5’-GGGCCAGGGAGGCCA-3’, 1 μL for DI*A allele detection and 5 μM of DI-AB-F forward primer, 1 μL and DI-B-R reverse primers 5’-GGGCCAGGGAGGCCG-3’, 1 μL for DI*B allele detection. Co-amplification of the human growth hormone (*HGH*) gene using 6 μM HGH forward primer 5’-TGCCTTCCCAACCATTCCCTTA-3’, 1 μL, and 6 μM HGH reverse primer 5’-CCACTCACGGATTTCTGTTGTGTTTC-3’; 1 μL was run as the internal control and 5 μL of PCR grade water. The PCR was performed with 10 μL of 2X PCR reaction mixture (OnePCR Plus, GeneDirex, Taiwan) in a G-STORM GS1 thermal cycler (Gene Technologies Ltd., Somerset, UK).

PCR was performed under the conditions described below, i.e., 95°C for 30 sec (initial denaturation). The cycle parameters of the PCR program began with the first step of 10 cycles of 30 sec at 95°C and 60 sec at 69°C, then 20 cycles of 10 sec at 95°C, 50 sec at 62°C and 30 sec at 72°C. The last step was final extension 5 min at 72°C and the sample was kept at 4°C. PCR products were electrophoresed at 100 volts with 1.5% agarose gel using 1X TBE buffer and were visualized under blue-light transilluminator. The PCR product size of both DI*A and DI*B alleles was 130 bp, whereas that of the internal control, the *HGH* gene was 434 bp.

Known DNA controls of Di(a+b-), Di(a+b+) and Di(a-b+) phenotypes were used as the controls. Altogether, 1,011 DNA samples of Thai blood donors were tested for DI*A and DI*B allele detection using PCR-SSP.

### DNA sequencing

Genomic DNA of 36 genotyped blood donors (one DI*A/DI*A, 20 DI*A/DI*B and 15 DI*B/DI*B) was sequenced to confirm the results of PCR-SSP. A fragment of 598 bp containing both SNPs (c.2561C/T) was obtained from PCR amplification of genomic DNA using the forward primer 5’-TTAGGGGTCCAGCTCACTCA-3’ and reverse primer 5’-TGACCGCATCTTGCTTCTGT-3’ and using similar PCR conditions to genotype DI*A and DI*B alleles.

### Statistical analysis

Gene frequencies were calculated by gene-counting method. The Chi-square (χ^2^) test was used to evaluate whether the observed genotype frequencies agreed with the expected ones under the Hardy-Weinberg equilibrium. The **χ**^2^ test of homogeneity was used to determine the difference between Central Thai and those previously reported in other populations including 9 Asians: Han Chinese (Shanghai) [24], Chinese (Shen-Zhen) [18], Chinese (Chengdu) [21], Brazilian Japanese descendants [16], Japanese [25], Korean (Seoul) [17], Korean [25], Southeast Asian [25] and Filipino [25] and 6 non-Asians: Southern Brazilians [15], Brazilians [20], Italians (Naples) [23], American Native [25], Alaska Native/Aleut [25] and Hawaiian/Pacific Islander [25], respectively. A P value equal or less than 0.05 was considered statistically significant. In addition, DI*A and DI*B allele frequencies were used to compute a matrix of Cavalli-Sforza chord distances for 9 Asian and 6 non-Asian populations [15–18, 20, 21, 23–25] by Phylip 3.695 [28]. A metric multidimentional scaling (MDS) analysis was performed using SPSS 16.0 software (SPSS Inc., Chicago, IL, USA) and was performed in different populations using the geometric genetic distances as parameters.

## Results

**Fig 1:**
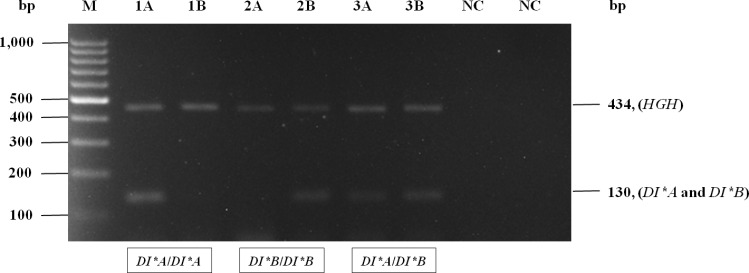
A representative gel showing Diego blood group genotyping by PCR-SSP technique. The 434 bp amplification product of the *HGH* control primer was present in all lanes, showing that amplification had occurred optimally. The genotype was deduced from the presence or absence of amplification products specific for DI*A (A), and DI*B (B) alleles. From left to right: Lanes 1A-1B = DI*A/DI*A; 2A-2B = DI*B/DI*B; 3A-3B = DI*A/DI*B and NC = negative control, respectively. Arrows indicate the size of *DI* gene fragments (right): DI*A and DI*B = 130 bp and internal control (*HGH*) = 434 bp. M: 100 bp ladder marker (Fermentas, Carlsbad, CA, USA).

The distribution of Di^a^ antigen among 391 Thai blood donors was studied. It was found that 13 of 391 (3.32%) were Di(a+) and 378 of 391 (96.68%) were Di(a-). Moreover, 1,011 DNA samples included 391 known Di^a^ antigen phenotype samples and an additional 620 samples were genotyped for DI*A and DI*B alleles by in-house PCR-SSP. The DNA controls were tested with two sets of primer combinations and the results agreed. According to the interpretation of PCR-SSP, homozygous DI*A/DI*A and DI*B/DI*B samples were positive with only the set of DI-AB-F and DI-A-R primers and the set of DI-AB-F and DI-B-R primers, respectively. In addition, heterozygous DI*A/DI*B samples were positive with both sets of primers, as shown in Fig 1.

**Fig 2:**
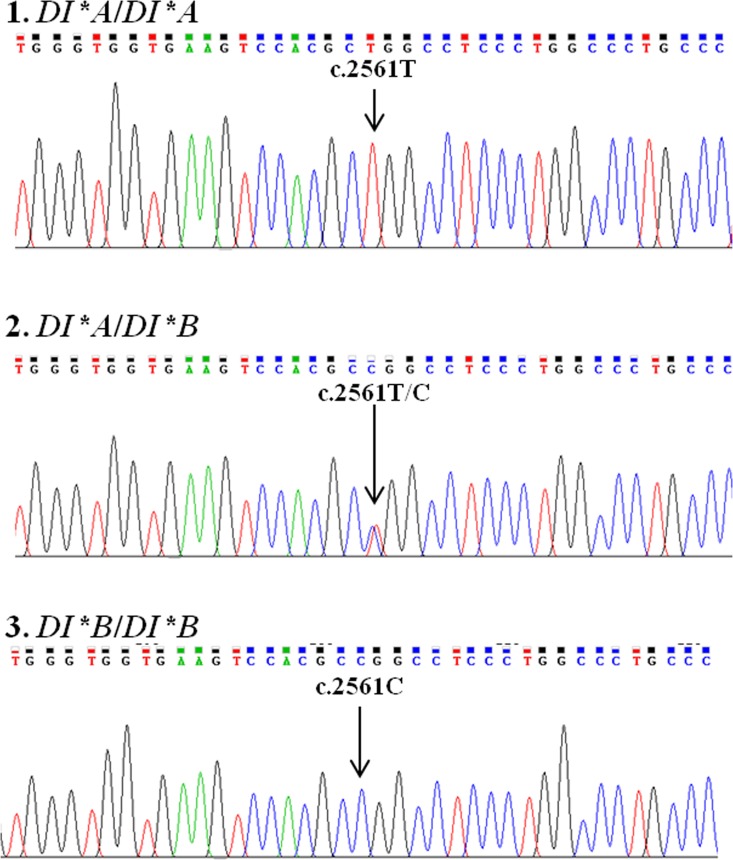
Electropherograms of the *DI* gene at the DI*A and DI*B polymorphism, SNP c.2561C>T. DNA sequences of the DI*A homozygote (1), the DI*A/DI*B heterozygote (2) and the DI*B homozygote (3) are amplified.

The results of DI*A and DI*B genotyping among 391 known Di^a^ antigen phenotype samples were computed to predicted phenotypes and were in agreement when compared with the phenotyping results by IAT. Additionally, 620 DNA samples were then tested for DI*A and DI*B genotyping using PCR-SSP. It was found that DI*B/DI*B was the most common (974/1,011), followed by DI*A/DI*B (37/1,011); whereas, DI*A/DI*A was not found in this study. Moreover, validity testing of the in-house PCR-SSP was performed using thirty-six randomly chosen DNA samples and the genotyping results showed 100% concordance between PCR-SSP and DNA sequencing (Fig 2).

The determined DI*A and DI*B genotypes among 1,011 Thai blood donors were consistent with the Hardy-Weinberg equilibrium (χ^2^ = 0.3535, P = 0.5527). Allele frequencies of DI*A and DI*B among Central Thais were 0.0183 (37/2,022) and 0.9817 (1,985/2,022), respectively. DI*A and DI*B allele frequencies between Central Thai and other populations were compared. Allele frequencies were similar to Southeast Asian, Brazilian, southern Brazilian and American native populations [15, 20, 25]. On the contrary, they significantly differed (P<0.05) from Italian, Alaska native/Aleut, Hawaiian/Pacific Islander, Han Chinese, Chinese, Japanese, Korean and Filipino populations [16–18, 21, 23–25], as shown in Table 1.

**Table 1:** Distribution of Diego blood group allele frequencies among different populations.

Population	Number	Allele frequencies		P value
		DI*A	DI*B	
**Central Thais**	1,011	0.0183	0.9817	
**Brazilians** [20]	4,326	0.0181	0.9819	0.9634
**Southeast Asian** [25]	942	0.0145	0.9855	0.3954
**American Native** [25]	970	0.0110	0.9890	0.0679
**Southern Brazilians** [15]	373	0.0282	0.9718	0.1454
**Filipino** [25]	1,333	0.0075	0.9925	0.0013^a^
**Chinese (Shen-Zhen)** [18]	1,766	0.0357	0.9643	0.0003^a^
**Alaska Native/Aleut** [25]	621	0.0065	0.9935	0.0077^a^
**Hawaiian/Pacific Islander** [25]	522	0.0060	0.9940	0.0083^a^
**Japanese** [25]	1,022	0.0430	0.9570	<0.0001^a^
**Brazilian Japanese descendants** [16]	209	0.0431	0.9569	0.0035^a^
**Han Chinese (Shanghai)** [24]	403	0.0447	0.9553	0.0001^a^
**Korean (Seoul)** [17]	116	0.0474	0.9526	0.0076^a^
**Korean** [25]	1,033	0.0580	0.9420	<0.0001^a^
**Chinese (Chengdu)** [21]	300	0.0600	0.9400	<0.0001^a^
**Italians (Naples)** [23]	225	0.0000	1.0000	0.0074^a^

^a^Significant differences from Central Thais (P<0.05; χ^2^ test)

**Fig 3:**
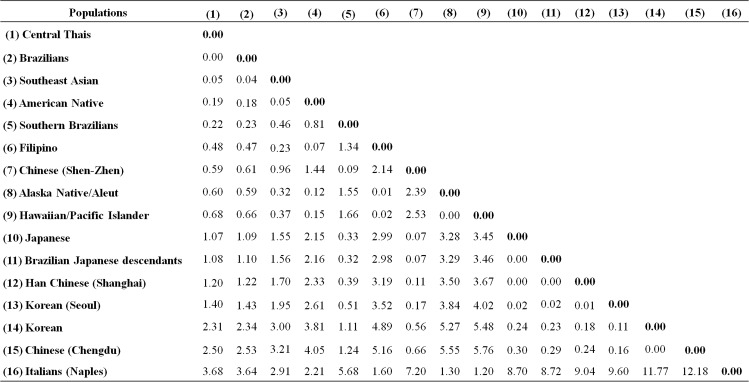
The pairwise matrix of geometric genetic distances of 16 populations based on DI*A and DI*B allele frequencies. The values are multiplied by 100. Arbitrary values were classified into three categories; highly related (0.00–0.40), moderately related (0.41–3.00) and unrelated (>3.01).

**Fig 4:**
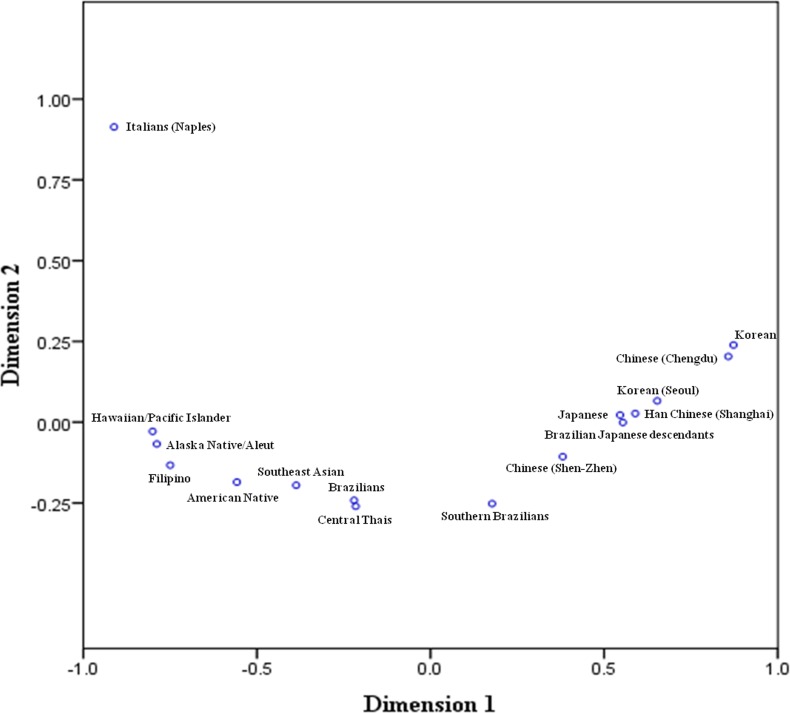
The metric multidimentional scaling analysis between Thais and other 15 populations.

Regarding an analysis of a matrix of geometric genetic distances, DI*A and DI*B allele frequencies of Central Thai blood donors were compared with 15 other blood donor populations examined in previous studies [15–18, 20, 21, 23–25]. Geometric genetic distances were calculated based on the allele frequencies of different populations. The pairwise matrix of geometric genetic distances of 16 populations is shown in Fig 3. The geometric genetic distances between Central Thais, Brazilian, Southeast Asian, American Native, and Southern Brazilian populations were highly related (<0.40); whereas, the distances between these populations and other populations including Filipino, Chinese (Shen-Zhen), Alaska Native/Aleut, Hawaiian/Pacific Islander, Japanese, Brazilian Japanese Descendant, Han Chinese (Shanghai), Korean (Seoul), Korean and Chinese (Chengdu) were moderately related (0.41–3.00). In addition, the distances between Central Thais and Italian (Naples) were compared and it was found that they were unrelated (>3.01). The metric MDS analysis of the 16 populations was performed based on allelic frequencies of the DI*A and DI*B as shown in Fig 4. Central Thais was found to be closest to those of Brazilians and Southeast Asia populations, and Italian (Naples) was placed at the furthest distance.

## Discussion

The prevalence of Di^a^ antigen is notably high among Asians of Mongoloid origin like Japanese, Chinese and Korean populations and the DI*A allele has never been found in unmixed European and African descendants [1]. The study of DI*A and DI*B alleles among populations can provide information for those working with anthropology and transfusion medicine [1, 13, 14]. In this study, the Di^a^ antigen phenotype was studied in 391 Central Thai blood donors and the prevalence of Di(a+) was 3.32%, similar to a recent study in the Thai population [29]. According to serological testing, ambiguous phenotypes between Di(a+b+) and Di(a+b-) cannot be excluded because anti-Di^b^ is not a marketed product. Moreover, in a previous study in Thailand, alloantibodies specific to the Diego blood group system, found in multitransfused patients were only anti-Di^a^ (1.2%) consisting of a single antibody (1.08%) and combined with other antibodies (0.12%) [30]. A positive DAT was often found among these patients; hence, genotyping can be used as an alternative to amend along with their limitations, especially in finding matched-blood donors for alloimmunized patients, assessing the risk of HDFN, and studying anthropology and disease associations [1, 13, 15, 16].

After DI genotyping using in-house PCR-SSP was implemented in 391 known Di^a^ antigen phenotype samples, the predicted phenotypes were in 100% concordance with the phenotyping results by IAT. Validity of in-house PCR-SSP for DI genotyping was performed and confirmed by DNA sequencing. The results of DNA sequencing were consistent with determined genotypes by PCR-SSP. Because the homozygous DI*A/DI*A alleles were not found among 1,011 Central Thais, finding extended-matched donors for a patient with anti-Di^b^ would be difficult. In this case, autologous or family-related donations are recommended. Moreover, screening for DI*A/DI*A individuals by PCR-SSP in a larger sample size may be helpful.

This is the first report of DI blood group allele frequencies in the Thai population. The DI*B allele was the most common and its frequency was similar to those observed among other populations [15–18, 20, 21, 23–25]. Concerning the matrix of geometric genetic distances, the arbitrary values of Central Thais were compared with other populations; it was found that the Central Thai population was highly related to Brazilian, Southeast Asian, American Native and Southern Brazilian populations [15, 20, 25]. One aspect in which Brazilian populations were highly related to the Central Thai population was because Brazil is heavily influenced by immigration and its population is ethnically very mixed. This should make its capacity for accepting migrants greater than that in very homogenous populations such as that of Japan. Even though the area and population size differ between Thailand and Brazil, both countries have tropical climates. In addition to international migration, creating a hybrid population through inter-marriage may be associated with genetic diversity [31]. Although Thailand and the Philippines are part of Southeast Asia geographically, the DI*A and DI*B allele frequencies are significantly different and geometric genetic distance showed that Filipino was moderately related to Central Thais. This may be due to the combined effects of group isolations and cultural practices such as within-group marriages that may have influenced those group differentiations [32]. However, one study using gene frequencies data of 29 genetic loci demonstrated that Thai and Filipino populations were much closer to each other than to European and African populations [33]. For the American native population, the DI*A and DI*B allele frequencies and geometric genetic distances were highly related to the Central Thai population. This may be because the analysis of a single gene is subjected large stochastic errors. Therefore, further studies of other relevant blood group alleles with larger sample sizes are required to confirm the relationships among populations.

Owing to the MDS analysis, East Asian populations including Japanese, Brazilian Japanese Descendant, Han Chinese (Shanghai), Korean (Seoul), Korean and Chinese (Chengdu) were moderately related to Central Thais and seemed to be all clustered together, which are consistent with population ethnicity and regions of residence. Notably, DI*A and DI*B allele frequencies, geometric genetic distances and MDS analysis of the Italian (Naples) populations were unrelated to the Central Thai and other Asian populations, similar to another study using the bootstrap tests with multiple genetic loci [33].

In conclusion, the similar prevalence of DI*A and DI*B alleles between Central Thai and Southeast Asian populations was demonstrated. A PCR-based identification of DI genotyping should overcome some of the serological limitations in transfusion medicine and provides a complementary tool for further population-genetic studies.
